# Congenital Lobar Emphysema

**Published:** 2009-08

**Authors:** Divya Chandran-Mahaldar, Subbaih Kumar, Kathamuthu Balamurugan, Arani R Raghuram, Rammaih Krishnan, Kannan

**Affiliations:** 1 Department of Cardiac Anaesthesiology, Meenakshi Mission Hospital and Research Center, Lakearea, Madurai TN 625107; 2 Department of Cardiothoracic and Vascular Surgery, Meenakshi Mission Hospital and Research Center, Lakearea, Madurai TN 625107; 3 Department of Pediatrics, Meenakshi Mission Hospital and Research Center, Lakearea, Madurai TN 625107

**Keywords:** Congenitallobar emphysema, Neonatal anaesthesia, Hyperinflation of emphysematous lung, Positive pressure ventilation

## Abstract

**Summary:**

Congenital lobar emphysema (CLE) characterized by over distension and air-trapping in the affected lobe is one of the causes of infantile respiratory distress requiring surgical resection of affected lobe.

At induction, positive pressure ventilation can expand the emphysematous lobe compressing the normal lung resulting in severe cardiovascular compromise. We report a case of 28 day old baby with CLE posted for emergency lobectomy. Strategies to prevent hyperinflation and anaesthetic considerations of various techniques adopted for lung separation in infants have been reviewed.

## Introduction

Congenital lobar emphysema (CLE) characterized by unilobar alveolar distension secondary to bronchomalacia or absent cartilage is a rare condition. This disease presents as respiratory distress due to the ventilation perfusion mismatch as a result of the hyper inflated lung causing compression atelectasis on the ipsilateral or the contralateral side with mediastinal shift. In 1954 Gross and Lewis, published the first case report of CLE.^1^ Male babies are affected more often than female in the ratio of 3:2.^2^. The incidence of left upper lobe involvement is 43%, right middle lobe 32%, right upper lobe 20%, and bilateral involvement 20%.^3^

The exact etiology of the disease is not known, but several intrinsic and extrinsic causes have been postulated 4. Presenting features in these infants can be dyspnea, tachypnea, retraction, wheezing, coughing, cyanosis, and asymmetric breath sounds. In these infants, there is increased intrathoracic pressure because of hyperinflation of one or more pulmonary lobes, leading to mediastinal shift and atelectasis of the ipsilateral or contralateral lobes of the lung. This causes displacement of heart sounds, decreased venous return, and varying degrees of ventilation-perfusion mismatch, which leads to hypoxia. Chest radiographs help to diagnose but is not definitive 4. A CT scan confirms the diagnosis and may rule out associated anomalous vascular slings. Associated congenital heart disease or vascular anomalies may occur in 12%-14% of these patients 5. Thus, all patients should have adequate preoperative cardiac evaluation by echocardiography and CT scan. Cardiac catheterization and angiography are necessary in children with known or suspected congenital cardiovascular lesions.

## Case report

A 28-day-old male baby weighing 3.4 Kg was referred as a case of perinatal asphyxia with respiratory distress not responding to medical management.

On examination, baby was tachypnoeic with flaring of alae nasi and sub costal retraction. The pulse rate was around 150/min. On examination of the respiratorysystem, decreasedbreath sounds on the left hemi thorax was noted. On examination of the cardiovascular system, the heart sounds were shifted to the opposite side. No gross cardiac anomalywas found.

**Fig 1 & 2:**
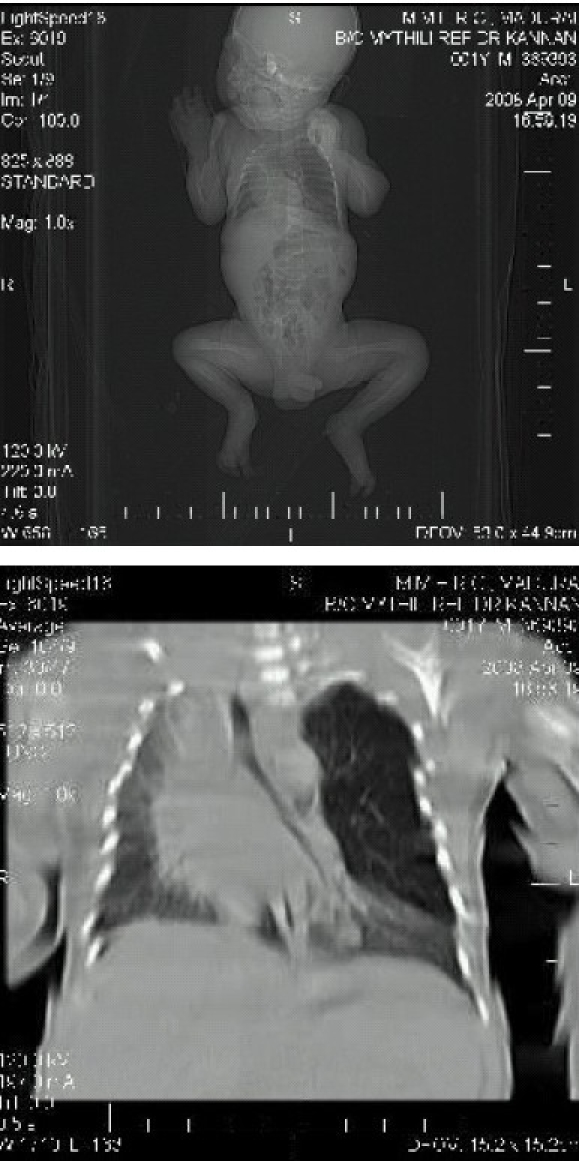
CT thorax showing hyperlucent, hyperexpanded left upper lobe causing compression of the remaining lung and mediastinal shift to right.

Following investigations were carried out: complete blood count, blood glucose, blood urea, serum creatinine and electrolytes and chest X-ray (PA and lateral view. Chest X-ray showed increased translucency onthe left side with tracheal and mediastinal shift to the right side. CT scan confumed the diagnosis of CLE of the left upper lobe (Fig 1,2).

The neonate was posted for left upper lobectomy.

### Anaesthetic management:

Preoperative examination revealed tachycardia and tachypnoea with signs of respiratory distress. On auscultation, there were decreased breath sounds on the left hemithorax. The cardiovascular system was normal. Oxygen saturation (SpO_2_) was 84% in air, but there was no visible cyanosis. Routine hematological and biochemical investigations were within normal limits. Echocardiography ruled out any associated congenital cardiac anomalies.

The baby was labeled as ASA III E. The baby was wrapped in warm cotton wool gamgees and placed on the heating mattress. Cardioscope and pulse oximeter was attached to the baby. Ryle's tube was aspirated with a syringe. Before starting anaesthesia, a surgeon was scrubbed to perform emergency thoracostomy if required.

Antisialogogue atropine 0.0lmg.kg^−1^ and fentanyl 3 mcg was given intravenously and rectal paracetamol suppository 80mg was placed. The baby was pre oxygenated for 5 minutes and then gradually sevoflurane was started. Gentle manual ventilation was performed via the facemask.

After introducing laryngoscope, a 3.5 size endotracheal tube was inserted. The baby was connected to anaesthesia machine through Jackson Rees modification of Ayre's ‘T-piece’.

Spontaneous ventilation was maintained using 100% oxygen, 1-2% sevoflurane with gentle manual ventilation. Saturation on pulse oximeter was 98% following intubation

The neonate was placed in true right lateral position

Monitoring included electrocardiogram, invasive blood pressure, SpO_2_, ETCO_2_ and rectal temperature. IV fluids were titrated according to Holiday Segar formula to replace fasting and maintenance requirements. Blood loss was replaced. Vital signs were maintained in normal range throughout surgery.

Once resection of the affected lobe was completed, controlled lung ventilation with atracurium as the neuromuscular blocking agent was started. Nitrous oxide was added thence.

Blood gases intraoperatively and postoperatively were within normal limits. At the end of operation, intercostal block was given with 3 ml of 0.125% bupivacaine, and residual neuromuscular block was reversed with neostigmine 0.15 mg along with atropine 0.03 mg IV. The infant was extubated when spontaneous respiration was sufficient to maintain SpO_2_>90% in air. Later, the child was kept in an oxygen-enriched environment in the pediatric intensive care unit under continuous SpO_2_ and EKG monitoring. At 72 hours the chest drain was removed after full expansion of the residual lung. Rest of the postoperative period was uneventful, and the child was discharged after 7 days.

**Fig 3:**
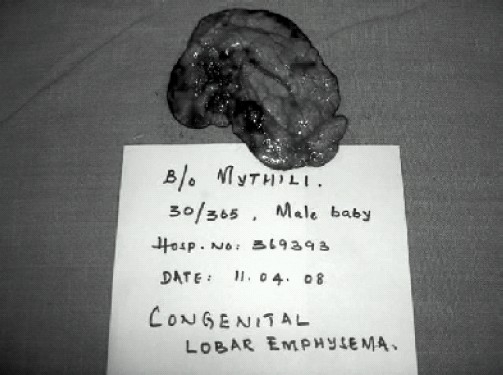
Gross specimen of the resected emphysematous left upper lobe.

**Fig 4:**
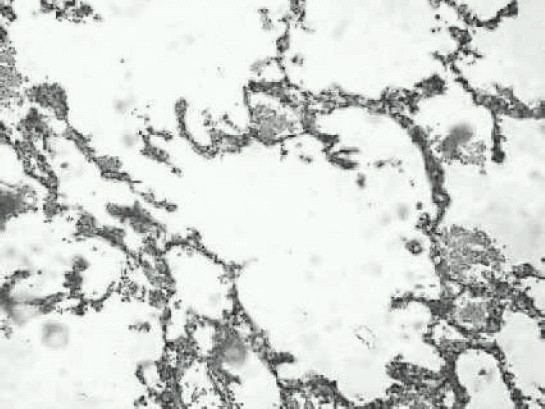
Microscopy showing distended alveolar spaces.

Lung biopsy of the resected segment showed lung parenchyma with atelectatic changes and emphysematous dilatation of alveolar spaces in the surrounding zone. (Fig 3,4).

## Discussion

Controversy still exists concerning the diagnosis and treatment of congenital lobar emphysema (CLE). Although surgical removal of the affected lobe is the most commonly accepted form of treatment, there is a small place for conservative therapy in patients who are not clinically inrespiratory distress and able to feed and grow. Maintaining ventilatory pressures and volume as low as possible avoids producing ventilatorrelated hyper expansion of the affected lobe. Management by more conservative, gentle ventilation technique if successful will result in fewer emergency surgeries with CLE. Operative mortality rate is 3 to 7% whereas with conservative therapies it is 50 to 75%. Hence conservative management should be reserved only for patients with milder symptoms or no distress at all. 6

Monitoring of the vital parameters, during neonatal surgery is a must. During thoracotomy, the baby is at great risk. On induction of anaesthesia if positive pressure ventilation is applied before opening of the chest, it may cause rapid inflation of emphysematous lobe or cyst with sudden mediastinal shift and cardiac arrest 7. Therefore, induction of anaesthesia should provide adequate spontaneous ventilation with minimal airway pressure. Occasional gentle assistance is necessary. Once the chest is opened and the affected lobe is delivered, the patient can be paralyzed and the lungs ventilated by controlled ventilation 8. Hyperinflation of the emphysematous lobe or cyst can be prevented by avoiding the use of nitrous oxide before the delivery of the affected lobe, as it diffuses faster in a closed cavity and expands the cavity, leading to further compression of normal lung andmore mediastinal shift^9^. In our patient nitrous oxide was started only after the affected lobe was resected.^10^,^11^

Isolated case reports of endobronchial intubation using single lumen endotracheal tube with gentle ventilation as an alternative to spontaneous bilaterallung ventilation are also described.^12^ However endobronchial intubation of the normal side leads to temporary collapse of the affected lobe with elimination of ventilation to the non perfused lung segment on the diseased side is an ever-present risk. Lack of double lumen tubes in this age group makes things difficult. Pediatric fibreoptic bronchoscope to confirm properplacement of the bronchial blocker was not available in our institute.

The technique of caudal thoracic epidural catheterization provides a stable cardiovascular profile and excellentanalgesia without depressing respiration. However kiniking and doubling back of catheter may prevent the catheter from reaching the midthoracic segments.^13^
